# Attitude of general dental practitioners, oral surgeons, and dentistry students towards oral lesions and oral biopsy

**DOI:** 10.4317/medoral.26804

**Published:** 2024-11-25

**Authors:** Verónica Schiavo-Di Flaviano, Beatriz González-Navarro, Carmen Martín Carreras-Presa, Enric Jané-Salas, José López-López

**Affiliations:** 1 ORCID 0000-0002-6295-4120. Master’s in Oral Medicine. Department of Odontostomatology. Faculty of Medicine and Health Sciences (Dentistry), University of Barcelona. Surgery and Implantology, Faculty of Medicine and Health Sciences (Dentistry), University of Barcelona, Barcelona, Spain; 2 ORCID 0000-0002-5951-7499. Department of Odontostomatology. Faculty of Medicine and Health Sciences (Dentistry), University of Barcelona - Oral Health and Masticatory System Group (Bellvitge Biomedical Research Institute) IDIBELL. University of Barcelona, Barcelona, Spain; 3 ORCID- 0000-0002-0937-0994.3. Oral Medicine Specialist. Faculty of Biomedical Sciences. Department of Dentistry. European University of Madrid. Madrid, Spain; 4 ORCID 0000-0002-3574-4603. Department of Odontostomatology. Faculty of Medicine and Health Sciences (Dentistry), University of Barcelona - Oral Health and Masticatory System Group (Bellvitge Biomedical Research Institute) IDIBELL. University of Barcelona, Barcelona, Spain; 5 ORCID 0000-0001-8035-4412. Department of Odontostomatology. Faculty of Medicine and Health Sciences (Dentistry). University of Barcelona - Clinical Chief of Odontological Hospital University of Barcelona - University of Barcelona Oral Health and Masticatory System Group (Bellvitge Biomedical Research Institute) IDIBELL. Barcelona, Spain

## Abstract

**Background:**

Oral biopsy is a fundamental surgical procedure used to obtain a histopathological result that assists clinicians in establishing a definitive diagnosis of oral mucosal lesions. The aim of this study was to asses the knowledge and attitudes of dental students, general dentists, and oral surgery experts regarding the detection of oral lesions and the use of biopsy as a diagnostic tool.

**Material and Methods:**

a self-administered questionnaire consisting of 26 questions and 3 clinical cases on oral lesions was distributed to general dentists, oral surgery specialists and final-year dentistry students at the University of Barcelona. A descriptive analysis of all variables included was performed. The chi-square test was used to compare categorical variables, and a multivariate logistic regression model was performed.

**Results:**

A total of 281 questionnaires were included in the study. In terms of diagnosing of oral lesions, 44.7% of students and 32.1% of general dentists were unable to make an accurate diagnosis, compared to 81.7% of specialists. Twelve students (15.8%) and twenty general dentists (14.9%) reported lacking the skills to perform a biopsy. Ninety general dentists (67.2%) reported feeling uncomforTable performing biopsies due to a lack of experience. Oral surgeons are 84.4 times more likely to identify lesions of the oral mucosa compared to students. General dentists experience 9.6 times more difficulty diagnosing oral lesions compared to students. General dentists are 0.43 times less likely to perform sample analysis compared to students.

**Conclusions:**

Oral biopsy is a procedure primarily performed by specialists in oral surgery, with its use among general dentists being limited, likely due to a lack of training in the field. To encourage the use of biopsy among general dentists, clinical training should be a fundamental component of the education of oral healthcare providers.

** Key words:**Oral biopsy, oral lesions, attitude of dentist, attitude of dental students.

## Introduction

Accurate diagnosis and treatment of oral diseases are essential components of oral health and can serve as indicators of a high-quality dental care. Oral mucosal lesions (OML) represent a wide spectrum of diseases that can present as isolated conditions or in association with dermatological diseases (1,2).

Approximately thirty percent of the general population may have OML (1), highlighting the importance of a thorough clinical history, examination of the oral mucosa, and proper patient assessment. These lesions can be benign, potentially malignant, or malignant, with early diagnosis being crucial for the latter two to reduce morbidity and mortality. Dentists' knowledge and skills are essential for establishing an accurate diagnosis and determining the appropriate course of treatment. Adequate training is required to ensure comprehensive knowledge of oral lesions in general and the early detection of oral cancer (3-6).

Different oral diseases may exhibit similar clinical features (2), making oral biopsy the Gold Standard for diagnosing OML when they are detected (2-4).

An oral biopsy is a surgical procedure in which a piece of tissue is removed from a living organism to be examined under a microscope to determine the lesion’s histological diagnosis (7). There are various classifications of biopsies based on their type, technique, type of lesion and location, materials used, timing of procedure, sample processing, and purpose of the biopsy. One of the most common classifications is whether the biopsy is incisional or excisional. In an incisional biopsy, a representative sample of the lesion is collected, whereas in an excisional biopsy, the entire lesion is removed along with safety margins of varying widths, depending on the initial diagnostic suspicion (8).

General dentists are trained to examine, diagnose, and treat a wide range of pathologies related to the oral cavity, including OML (2). However, some clinicians, such as oral surgeons, are more familiar with the oral mucosa and surgical procedures, leading to ongoing debate about whether the diagnosis of oral soft tissue diseases should be performed by general dental practitioners (GDPs) or reserved for specialists (2,3).

Currently, there is limited evidence available regarding the attitudes and knowledge of dentists concerning oral mucosa lesions. This study aims to assess the attitudes and knowledge of dental students, general dental practitioners (GDPs), and oral surgery specialists regarding their awareness of oral mucosa lesions and the use of oral biopsy as a diagnostic method.

## Material and Methods

- Study design and ethical considerations

A cross-sectional study using a self-administered questionnaire was approved by the Ethics and Drugs Committee of the University of Barcelona Dental Hospital (CEICm-HOUB) (Protocol ID: 38/2017). Upon reasonable request, the data presented in this article may be available by the Correspondence. Informed consent was obtained from all participants. This research was conducted in accordance with the Declaration of Helsinki (9).

**Figure 1:**
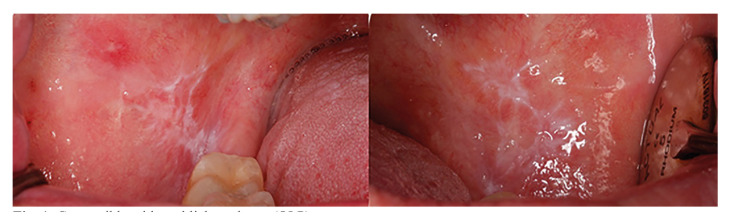
Compatible with oral lichen planus (OLP).

**Figure 2:**
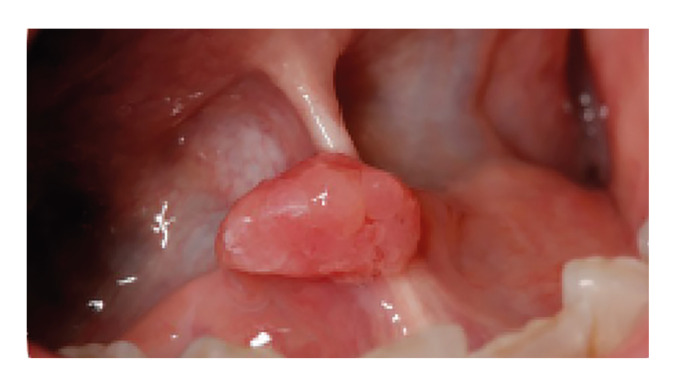
Compatible with papilloma.

**Figure 3:**
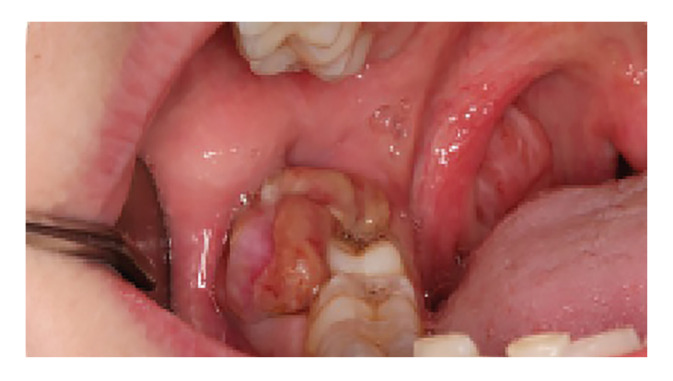
Compatible with oral squamous cell carcinoma (SCC).

The questionnaire consisted of 26 questions and three images. Questions 1-6 focused on demographic data and the level of training of the participants; questions 7 and 8 addressed mucosal examination; questions 9-13 pertained to oral lesion detection; questions 14-19 explored attitudes toward oral lesions and biopsy; questions 20-22 gathered opinions regarding oral biopsy as a diagnostics and therapeutic procedure; and questions 23-26 examined attitudes toward oral mucosa lesions from a therapeutic standpoint. The three clinical images represented an oral lichen planus (Fig. 1), a squamous papilloma on the lingual frenulum (Fig. 2) and a lesion suspicious for oral squamous cell carcinoma (Fig. 3).

Three questions were posed in each case: the most likely clinical diagnosis, whether the professional would perform a biopsy to diagnose the lesion, and what type of biopsy was indicated. The interviewees responded to the closed-ended questions by selecting the option they considered most accurate and relevant to the situation.

A pilot survey was conducted beforehand and distributed to 15 dental professionals, selected based on their accessibility and proximity to the research team, to ensure that the questions were appropriate, comprehensible, and accepTable among professionals. Three oral medicine experts from the European University of Madrid, the University of Santiago de Compostela, and the University of Seville were asked to evaluate the clinical cases. They unanimously agreed that the images accurately depicted the pathologies that needed to be diagnosed. The Kappa-Cohen agreement for the three professors was 1 for a score of 9.5-10 on the questionnaire. The three groups in the pilot study were analyzed for agreement for a response greater than 8 on the questionnaire, the results were 0.8 for students and general dental practitioners and 1 for oral surgeons.

- Sample size

The sample size was determined to assess the percentage of individuals with knowledge about potentially malignant oral lesions (92.3%) and the prevalence of professionals knowledgeable about the indications for biopsy (7.8%), based on the findings of Anandani *et al* (1). The sample size calculation was based on a 95% confidence level, a precision level of 5% and a non-response rate of 30%. The largest estimated sample size was 236 participants. A convenience sampling was employed, with a minimum of 50 participants per group.

- Participants recruitment and inclusion/exclusion criteria

The survey was personally distributed to dental students in their final year at University of Barcelona, as well as to general practitioners and oral surgery specialists working in various private practice centers across the city of Barcelona.

Distribution of the questionnaire occurred between November 2022 and November 2023. The surveys were completed anonymously, with respondents placing their questionnaires in a sealed container to ensure anonymity. The containers were not unsealed until December 2023.

The study initially included all participants who responded to the questionnaire. However, individuals who did not provide information on age, gender, or professional level were excluded from the study after the data was collected.

- Statistical analysis

Data were collected using the Excel Corporation program (Microsoft, Redmond, WA). All analyses were performed with the SPSS Statistics program (Version 12, SPSS Inc, Chicago, IL). A descriptive analysis was conducted for all included variables. Numerical variables were described based on their distribution as mean and standard deviation or as median, minimum, maximum and 25th and 75th percentiles (P25-P75). Categorical variables were described using frequencies and percentages.

The chi-square test was used to compare categorical variables. A multivariate logistic regression model, adjusted for age, sex, year of dental degree, and approximate number of weekly visits, was used to assess whether there were differences among dental professionals (degree of training) regarding knowledge of potentially malignant oral lesions and attitude towards biopsy. The measure of association was the Odds Ratio (OR) which was reported with a 95% confidence interval. A *p-value* of <0.05 was considered statistically significant.

## Results

The survey was distributed to 76 fifth-year dentistry students (27%), 134 general dental practitioners (GDPs) (47.7%), and 71 oral surgery specialists (25.3%). The response rate was 100%, resulting in a total sample of 281 participants with no surveys needing to be discarded.

The final sample included 103 men (36.7%) and 178 women (63.3%), with a mean age of 30.07 years and an average of 5.85 years of experience. The participants reported caring for an average of 68 patients per week. (Table 1).

Regarding the question on routine examination of oral mucosa, 56 students (73.7%) and 66 (93%) specialists reported performing it routinely, while only 69 GDPs (51.5%) indicated that they regularly perform this examination (Table 2).

Regarding the identification, diagnosis, and treatment of oral lesions, 34 students (44.7%) and 43 GDPs (32.1%) reported not feeling confident in their diagnostic abilities, in contrast to 58 specialists (81.7%) who expressed confidence in their diagnoses. Additionally, 53 GDPs (39.5%) admitted to having difficulties when diagnosing oral lesions, while 86 (60.45%) reported encountering difficulties only occasionally (Table 2).

Concerning knowledge of the biopsy technique (Table 2), 15.8% of the students and 14.9% of GDPs stated that they do not know how to perform a biopsy procedure. While 97.2% of specialist reported having mastered the technique, 60.5% of students and 41.0% of GDPs believed their knowledge was limited.

Table 2 displays the answers to questions 16, 17, 19, and 24 as well. Considering all three groups, 47.3% of participants reported identifying a lesion at least once a year, 21.0% reported detecting a lesion at least once a month, and 14.2% reported never detecting a lesion; of these, the majority were GDPs (15.7%) and students (23.7%). In terms of attitudes toward lesions that need to be biopsied, 100% of students and 73.1% of GDPs refer all cases to a specialist, while 43.7% of specialists only refer cases involving large or suspected malignant lesions, or those with difficult localization to maxillofacial surgeons at public hospitals. According to the responses to question 19, 71.1% of students had never performed a biopsy, while 28.2% of students had done at least one biopsy within the previous 2 years. In comparison, 67.2% of GDPs do not perform biopsies, whereas 42.3% and 38% of specialists perform biopsies at least once a year or once a month, respectively.

To determine the main reasons why GDPs do not perform biopsies, question 18 was included in the questionnaire. The most common responses were lack of experience (59.7%), lack of resources needed to perform biopsies (32.1%), lack of knowledge (29.1%), and lack of confidence in the interpretation of the results (27.6%).

A question was added to the survey to determine whether participants sent the biopsy sample for analysis after the procedure (Table 2). Among GDPs, 21.6% only occasionally sent samples for analysis, and 20.15% did not send any samples at all. In contrast, 59 oral surgeons (83.10%) and 60 students (78.9%) consistently sent samples to the pathologist.

Table 2 also presents participants’ opinions on who should perform biopsies. 40.8% of students, 47.01% of GDPs and 39.4% of specialists considered that GPD´s should only perform “simple” biopsies and those involving benign-looking lesions.

After adjusting the Odds Ratio for age, sex, years of experience and number of weekly visits, routine mucosal examination was conducted 6.7 times more frequently by oral surgeons (OR=6.7, 95% CI [2.06-21.7]) and 0.5 times less frequently by GDPs compared to students (OR=0.5, 95% CI [0.22-0.99]). Additionally, oral surgeons were 84.4 times more likely to detect lesions of the oral mucosa compared to students (OR=84.4, 95% CI [22.9-312.3]) (Table 3).

GDPs have 9.6 times greater difficulty diagnosing oral lesions compared to students (OR=84.4, 95% CI [22.9-312.3]). Oral surgeons possess 118.9 times more knowledge of the biopsy technique (OR=118.9, 95% CI [23.9-590.2]), while GDPs only 2.6 times more knowledge compared to students (OR=2.6, 95% CI [1.22-5.68]) (Table 3). Additionally, GDPs are 0.43 less likely to send samples for analyses than students (OR=0.43, 95% CI [0.20-0.92]) (Table 3). GDPs are 2.6 times more likely to treat OML than students (OR=2.6, 95% CI [1.2-6.8]), and specialists are 9.6 times more likely to do so (OR=9.6, 95% CI [3.2-29.1] ) (Table 3).

Regarding clinical case 1 (images corresponding to oral lichen planus), 70.9% of GDPs and 91.5% of oral surgeons correctly diagnosed the lesions in the image, while 34.2% of students failed to make the correct diagnosis. In terms of their approach to this case, 64.9% of GDPs and 91.5% of oral surgeons would perform a biopsy to confirm the diagnosis, with the majority believing that an incisional biopsy was indicated. On the other hand, 60.5 % of students would not perform the biopsy, as they believed it was not indicated (Table 4). There were statistically significant differences in accuracy of the diagnosis between the groups compared (*p*<0.001), with greater differences between oral surgeons and students, and no statistically significant differences between GDPs and specialists. Regarding the indication of a biopsy to confirm diagnosis, there were statistically significant differences across all groups analyzed (*p* <0.005) (Table 4).

For the diagnosis of clinical case 2 (image compatible with papilloma), 64.2% of GDPs, 60.6% of oral surgeons, and 76.3% of students made an incorrect diagnosis. However, 95.5% of GDPs, 90.1% of oral surgeons, and 94.7% students agreed that a biopsy of this lesion was necessary and that it should be excisional. (Table 4). In this case, statistically significant differences were found in terms of a correct diagnosis between students and GDPs (*p* = 0.02) and between students and oral surgeons (*p* <0.001), but there were no significant differences between GDPs and oral surgeons (*p* = 0.07) (Table 4).

A total of 15.7% of GDPs and 15.8% of students made an incorrect diagnosis in the third clinical case (a clinical picture consistent with squamous cell carcinoma), compared to just 1.4% of oral surgeons. Regarding the biopsy procedure, the findings among the three groups were comparable: 63.4% of GDPs, 73.2% of specialists, and 63.2% of students stated that they would biopsy the lesion. Table 4 shows that 19.7% of students believed that a biopsy was not necessary in this case, while 27.61% of GDPs were unsure about the type of biopsy that should have been performed. Statistically significant differences were observed in the accuracy of diagnosis between GDPs and oral surgeons (*p* = 0.001) (Table 4).

## Discussion

Biopsy is a crucial procedure due to its role in the diagnosis and early detection of oral cancer. Although identifying lesions compatible with oral cancer can be challenging, it is essential that professionals are capable of recognizing abnormal structures in the oral cavity and taking appropriate actions for diagnosis and treatment (10,11). A fundamental aspect of diagnosing these lesions is the examination of the oral mucosa. In our survey, only 51.49% of GDPs reported performing routine oral mucosa examinations. This finding aligns with the results from a questionnaire conducted in Yemen (12), where only 68.30% of respondents stated they routinely performed oral mucosa examinations, and with another study carried out in The Netherlands (13), where 65.9% of participants reported inspecting the oral mucosa in all patients.

We would like to emphasize that 32.09% of GDPs in our study reported not diagnosing oral lesions, attributing their difficulties primarily to lack of experience and insufficient undergraduate training. This reported lack of experience is consistent with findings from other studies (5), where the majority of professionals preferred to refer OML cases to specialists or higher centers.

Various studies report a low percentage of GDPs who utilize biopsy as a diagnostic method. This is evident in the results of Diamanti *et al*. (14) in 2002, where only 15% of GDPs had performed biopsies in the previous two years, while 55% preferred to refer patients with oral lesions to specialists. In the first study conducted in Spain by López Jornet *et al*. (15) in 2007, which focused exclusively on general dentists, 32.1% used biopsy as a diagnostic method, and 52.8% preferred to refer the patients to specialists. Other studies similarly demonstrate low percentages of general dentists who have performed or routinely perform biopsies, with Figures such as 6.66% (16), 22.7% (17), 28.7% (18), 29.5% (5), and 34.4% (19). A study published in 2023 (20) conducted among dental students and dentists in Brazil found that 7.9% of the participants never performed biopsies, and 61,8% rarely did so. Our study yielded similar results, with 67.16% of GDPs not performing biopsies and 73.13% referring all cases to specialists.

Several authors have used portions of their questionnaire to explore the reasons why GDPs do not perform biopsies. The main reasons reported included lack of experience, fear of making a diagnostic error, insufficient knowledge to interpret the pathologist's report, or concern about obtaining a non-representative sample of the lesions (9,14,15,17,20).

The lack of necessary instruments (9,14,15,20) was also a frequent concern. To a lesser extent, other concerns included the fear of legal implications (14,17), the possibility of intraoperative emergencies (17), lack of access to a nearby histopathology laboratory (1), dissemination of tumor cells, and insufficient financial compensation for the procedure (14). In our study, the primary reasons for GDPs not performing biopsies in their daily practice were a lack of experience (56.23%) and insufficient means to perform a biopsy (26.33%).

Additionally, there is a significant difference between GDPs and specialists regarding the performance of biopsies. Similar results were observed by Wan & Savage (17), where only 22.7% of GDPs performed biopsies compared to 73.7% of specialists. It is important to note that this study did not differentiate between types of specialties. In contrast to our findings regarding specialists performing biopsies, only 7.5% of specialists in the study by Shrestha & Subedi (21) performed biopsies, almost ten times less than in our study. These results could be related to the fact that the majority of specialist in their study where between 25-30 years old and had reported less than five years of experience.

As a possible measure to achieve more consistent results between both groups, it would be advisable to implement continuing training courses. This suggestion is supported by the findings of Anandani *et al* (11), where all surveyed professionals expressed a need to update their knowledge about oral mucosa lesions and the performance of biopsies.

There are varying opinions on whether general dentists should perform biopsies. In our study, less than half of the professionals believe that this procedure should be part of the general dental practice. According to Warnakulasuriya & Johnson (22), only 21% of the GDPs surveyed would take samples of suspicious lesions, whereas maxillofacial surgeons prefer to receive lesions without any alterations caused by scarring from a prior biopsy. Photographic documentation of lesions before biopsy could serve as a useful tool, particularly in cases where the referral is made after the biopsy has been performed.

Supporting these findings, the study by Diamanti *et al*. (14) revealed that 70% of the maxillofacial surgeons surveyed would discourage general dentists from performing biopsies, with only 30% believing that general dentists should be able to perform simple biopsies, preferably excisional and limited to benign lesions; the main concern among surgeons was the lack of technical expertise and the inability of GDPs to take a representative sample of the lesion, with the major concern of a possible delay or misdiagnosis of serious pathology. In contrast, a study conducted in Spain by Seoane *et al*. (23) found that 84.4% of dentists consider biopsy a surgical procedure that should be routinely used in clinical practice.

Regarding the clinical case of oral lichen planus (Case 1), a higher percentage of correct diagnoses and indicated biopsies were observed as the clinician’s experience increased.

In the clinical case of squamous papilloma (Case 2), students had a significantly lower percentage of correct diagnoses compared to specialists, but there was a higher indication for biopsy than in other clinical cases. We believe that the atypical location of this lesion may have generated greater uncertainty in the diagnosis, making a biopsy a necessary procedure to achieve an accurate diagnosis.

In the case of oral carcinoma (Case 3), similar results were observed across all three groups in terms of diagnosis, with biopsy indicated in similar proportions. Based on our findings, we can infer that as the suspicion of malignancy and diagnostic uncertainty increase, so does the indication for biopsy. For lesions that do not generate diagnostic doubts, biopsies are more frequently indicated by specialists, possibly due to their extensive knowledge of the diagnostic and therapeutic process for such lesions.

One of the main limitations of the study is that it is not a questionnaire validated by the scientific community and although it has been evaluated by three professors and a pilot study has been carried out to assess the relevance of the questions, the conclusions must be taken with caution. In addition, we have focused on only three images and the population is from a single center.

## Conclusions

We conclude that oral biopsy is a procedure practiced by specialists in oral surgery and oral medicine, with its use among general dentists being limited. According to our results, this limitation is largely due to a lack of experience and training in the field.

General dentists are more likely to indicate biopsies in more difficult cases, and the more experienced the clinicians are, the more likely they are to use biopsy as a diagnostic method.

To encourage the use of biopsy among general dentists, greater emphasis should be placed on undergraduate education and continuing educations courses. As suggested by the participants, there should be a stronger focus on training students in the detection of oral mucosa lesions, the appropriate indication for biopsy, and the acquisition of the necessary skills to perform the procedure.

## Figures and Tables

**Table 1:** Demographics and sample distribution.

Variables		Total (N=281)	Students (N=76)	GDPs (N=134)	Specialists (N=71)
Age	Mean (DE)	30,07 (8.6%)	23,88 (2.10%)	31,35 (7.25%)	34,30 (10.25%)
Gender	Men (%)	103 (36.7%)	21 (27.6%)	51 (38.1%)	31 (43.7%)
	Women (%)	178 (63.3%)	55 (72.4%)	83 (61.9%)	40 (56.3%)
Years since completion of studies	Mean (DE)	5,85 (6.78%)	0 (0.00%)	7,04 (6.29%)	9,85 (7.28%)
Number of weekly visits	Mean (DE)	68,00 (73.61%)	8,25 (10.34%)	85,48 (79.01%)	98,99 (65.30%)

**Table 2:** Answers to the questions 7, 9, 10, 12, 14, 15, 16, 17, 19, 20, 21, 22, 23, 24 and questions included in clinical case 1, 2 and 3.

Question		Students	GDPs	Specialists	P-Value		
					Students vs GDP	Students vs Specialist	GDP vs specialists
P-7. Routinary exploration of oral mucosa	Yes	56 (73.7%)	69 (51.5%)	66 (93.0%)	0,0017^1^	0,0018^1^	<0,0001^1^
	No	1 (1.3%)	16 (11.9%)	2 (2.8%)			
	Sometimes	19 (25.0%)	49 (36.6%)	3 (4.2%)			
P-9. Frequency in which Oral mucosal lesions are found	Never	0	10 (7.5%)	1 (1.4%)	<0,0001^1^	<0,0001^1^	<0,0001^1^
	Once in 2 years	10 (13.5%)	23 (17.2%)	6 (8.5%)			
	Once a year	60 (81.1%)	48 (35.8%)	3 (4.2%)			
	Once a month	2 (2.7%)	43 (32.1%)	34 (47.9%)			
	Once a week	2 (2.7%)	10 (7.5%)	22 (31.0%)			
	More than once a week	0	0	5 (7.0%)			
	No answer	2	0	0			
P-10. Diagnosis of oral mucosal lesions	Yes	15 (19.7%)	33 (24.6%)	58 (81.7%)	0,1879	<0,0001^1^	<0,0001^1^
	No	34 (44.7%)	43 (32.1%)	3 (4.2%)			
	Sometimes	27 (35.5%)	58 (43.3%)	10 (14.1%)			
P-12. Difficulties in the diagnosis of oral mucosal lesions	Yes	42 (55.3%)	53 (39.5%)	4 (5.6%)	0,0100	<0,0001^1^	<0,0001^1^
	No	2 (2.6%)	0	21 (29.6%)			
	Sometimes	32 (42.1%)	81 (60.4%)	46 (64.8%)			
P-14. Referral of oral mucosal lesions to the specialists	Yes	59 (77.6%)	101(75.4%)	33 (46.5%)	0,4351	<0,0001^1^	<0,0001^1^
	No	2 (2.6%)	9 (6.7%)	18 (25.4%)			
	Sometimes	15 (19.7%)	24 (17.9%)	20 (28.2%)			
	Yes	18 (23.7%)	59 (44.0%)	69 (97.2%)			
P-15. knowledge about biopsy techniques	No	12 (15.8%)	20 (14.9%)	0	0,0094	<0,0001^1^	<0,0001^1^
	Yes, but scarce	46 (60.5%)	55 (41.0%)	2 (2.8%)			
P-16. Frequency in which you find lesions that require biopsy	Never	18 (23.7%)	21 (15.7%)	1 (1.4%)	<0.0001^1^	0.0003^1^	<0.0001^1^
	Once in 2 years	14 (16.4%)	29 (21.6%)	1 (1.4%)			
	Once a year	44 (57.9%)	58 (43.3%)	31 (43.7%)			
	Once a month	0	26 (19.4%)	33 (46.5%)			
	Once a week	0	0	5 (7.0%)			
P-17. Do you perform the biopsy?	Refer all	76 (100.0%)	98 (73.1%)	9 (12.7%)	<0.0001^1^	<0.0001^1^	<0.0001^1^
	Yes, only benign	0	4 (3.0%)	10 (14.1%)			
	Yes, all kind	0	6 (4.5%)	21 (29.6%)			
	Referral of difficult cases	0	26 (19.4%)	31 (43.7%)			
P-19. Frequency in which you perform a biopsy	I don't biopsy	54 (71.1%)	90 (67.2%)	11 (15.5%)	<0.0001^1^	<0.0001^1^	<0.0001^1^
	Once in two years	22 (28.9%)	12 (9.0%)	3 (4.2%)			
	Once a year	0	20 (14.9%)	30 (42.3%)			
	Once a month	0	12 (9.0%)	27 (38.0%)			
P-20. You believe that biopsy performance is:	Essential	24 (31.6%)	41 (30.6%)	42 (59.2%)	0.0002^1^	0.8019	0.0013^1^
	Important	42 (55.3%)	79 (59.0%)	27 (38.0%)			
	Mildly important	10 (13.2%)	14 (10.4%)	2 (2.8%)			
P-21. Do you believe GDPs should perform oral biopsies?	Yes, any kind	45 (59.2%)	52 (38.8%)	35 (49.3%)	0.3495	0.0004^1^	0.0098
	No	0	19 (14.2%)	8 (11.3%)			
	Only simple and benign	31 (40.8%)	63 (47.0%)	28 (39.4%)			
P-22. Do you believe the analysis of the sample is:	Essential	63 (82.9%)	60 (44.8%)	66 (93.0%)	<0.0001^1^	<0.0001^1^	0.1506
	Important	12 (15.8%)	59 (44.0%)	5 (7.0%)			
	Mildly important	1 (1.3%)	15 (11.2%)	0			
P-23. Do you analyze the samples?	Yes	60 (78.9%)	78 (58.2%)	59 (83.1%)	0.0004^1^	0.0019^1^	0.8065
	No	3 (3.9%)	27 (20.1%)	2 (2.8%)			
	Sometimes	13 (17.1%)	29 (21.6%)	10 (14.1%)			
P-24. Do you treat the lesion?	Yes	11 (14.9%)	28 (20.9%)	29 (40.8%)	<0.0001^1^	0.0369	<0.0001^1^
	No	51 (68.9%)	68 (50.7%)	4 (5.6%)			
	Sometimes	12 (16.2%)	38 (28.4%)	38 (53.5%)			
	No answer	2	0	0			
C1.1 OLP Diagnostic	Right	50 (65.8%)	95 (70.9%)	65 (91.5%)	0.4418		0.0007^1^
	Wrong	26 (34.20%)	39 (29.1%)	6 (8.5%)					
C1.2 OLP Biopsy	Yes	30 (39.5%)	87 (64.9%)	65 (91.5%)	0.0004^1^		<0.0001^1^
	No	46 (60.5%)	47 (35.1%)	6 (8.5%)					
C1.3 OLP Biopsy technique	Incisional	28 (36.8%)	73 (54.5%)	63 (88.7%)	0.014		<0.0001^1^
	Excisional	0	5 (3.7%)	3 (4.2%)					
	I don't know	2 (2.6%)	19 (14.2%)	0					
	Not indicated	46 (60.5%)	37 (27.6%)	5 (7.0%)					
C2.1 OP Diagnostic	Right	18 (23.7%)	48 (35.8%)	28 (39.4%)	0.0687		0.6101
	Wrong	58 (76.3%)	86 (64.2%)	43 (60.6%)					
C2.2 OP Biopsy	Yes	72 (94.7%)	128 (95.5%)	64 (90.1%)	0.7973		0.1325
	No	4 (5.3%)	6 (4.5%)	7 (9.9%)					
C2.3 OP Biopsy technique	Incisional	13 (17.1%)	11 (8.2%)	2 (2.8%)	0.0019^1^		0.0042^1^
	Excisional	47 (61.8%)	109 (81.3%)	68 (95.8%)					
	I don't know	12 (15.8%)	14 (10.5%)	1 (1.4%)					
	Not indicated	4 (5.3%)	0	0					
C3.1 SCC Diagnostic	Right	64 (84.2%)	113 (84.3%)	70 (98.6%)	0.982		0.0017^1^
	Wrong	12 (15.8%)	21 (15.70%)	1 (1.4%)					
C3.2 SCC Biopsy	Yes	48 (63.2%)	85 (63.4%)	52 (73.2%)	0.9683		0.1559
	No	28 (36.8%)	49 (36.6%)	19 (26.8%)					
C3.3 SCC Biopsy technique	Incisional	41 (53.9%)	60 (44.8%)	50 (70.4%)	0.2012		0.0005^1^
	Excisional	11 (14.5%)	18 (13.4%)	14 (19.7%)					
	I don't know	9 (11.8%)	37 (27.6%)	4 (5.6%)					
	Not indicated	15 (19.7%)	19 (14.2%)	3 (4.2%)					

OLP= Oral lichen planus; OP= Oral papilloma; SCC= Squamous cell carcinoma. ^1^Statistically significant differences between and each of the compared groups (p<0.005).

**Table 3:** Attitude towards oral mucosal lesions and Attitude toward oral biopsy performance and Attitude towards oral lesion treatment.

Attitude	Profession	OR crude (IC 95%)	OR adjusted (IC 95%)
Routinary exploration of oral mucosa ^1^	Specialists	4.7 (1.66-13.4)	6.7 (2.06-21.7)
	GDPs	0.4 (0.21-0.70)	0.5 (0.22-0.99)
	Students	1	1
Frequency in which Oral mucosal lesions are found ^2^	Specialists	106.7 (31.8-357.7)	84.4 (22.9-312.3)
	GDPs	11.4 (3.9-33.2)	9.4 (2.97-29.5)
	Students	1	1
Diagnosis of oral mucosal lesions^ 3^	Specialists	18.1 (7.95-41.4)	24.3 (8.73-67.9)
	GDPs	1.3 (0.67-2.64)	1.6 (0.74-3.71)
	Students	1	1
Difficulties in the diagnosis of oral mucosal lesions ^4^	Specialists	0.06 (0.01-0.29)	0.13 (0.02-0.86)
	GDPs	3.6 (0.32-40.3)	9.6 (0.59-155.6)
	Students	1	1
Referral of oral mucosal lesions to the specialists ^5^	Specialists	1	1
	GDPs	4.7 (1.99-11.2)	4.6 (1.79-11.9)
	Students	12.6 (2.79-56.5)	4.5 (0.83-24.8)
Knowledge about biopsy techniques ^1^	Specialists	111.2 (24.7-499.2)	118.9 (23.9-590.2)
	GDPs	2.5 (1.4-4.7)	2.6 (1.22-5.68)
	Students	1	1
Frequency in which you perform biopsy ^2^	Specialists	Not estimable	Not estimable
	GDPs	Not estimable	Not estimable
	Students	Not estimable	Not estimable
Do you send the sample to the pathologist? ^3^	Specialists	1.3 (0.57-3.01)	1.7 (0.63-4.52)
	GDPs	0.4 (0.19-0.71	0.43 (0.20-0.92)
	Students	1	1
Do you treat the lesion?	Specialists	3.9 (1.8-8.8)	9.6 (3.2-29.1)
	GDPs	1.5 (0.7-3.2)	2.6 (1.02-6.8)
	Students	1	1

^1^Answer: yes, always; ^2 ^Answer: at least once a month; ^3 ^Answer: yes; ^4 ^Answer: yes or occasionally; ^5 ^Answer: yes or sometimes. Odds Ratio Adjusted by age, sex, years of experience and number of seminal visits.

**Table 4:** Relationship between correct diagnosis and indication of biopsy according to type of professional.

Diagnosis	Profession	Right diagnosis	Biopsy performance	Right diagnosis and biopsy indication
OLP	Students (n=76)	50 (65.8%)^1^	30 (39.5%)^2^	30 (60%)^2^
	GDPs (n=134)	95 (70.9%)^1^	87 (64.9%)^2^	78 (82.1%)^2^
	Specialists (n=71)	65 (91.5%)^1^	65 (91.5%)^2^	60 (92.3%)^2^
Papilloma	Students (n=76)	18 (23.7%)^3^	72 (94.7%)	18 (100%)
	GDPs (n=134)	48 (35.8%)^3^	128 (95.5%)	48 (100%)
	Specialists (n=71)	28 (39.4%)^3^	64 (90.1%)	28 (100%)
SCC	Students (n=76)	64 (84.2%)	48 (63.2%)	46 (71.9%)
	GDPs (n=134)	113 (84.3%)^4^	85 (63.4%)	83 (73.5%)
	Specialists (n=71)	70 (98.6%)^4^	52 (73.2%)	52 (74.3%)

^1^There are differences between the compared groups (p<0.001), however, there are no statistically significant differences regarding the correct diagnosis between dentists and specialists. ^2^Statistically significant differences between and each of the compared groups (p<0.005). ^3^Statistically significant differences regarding the correct diagnosis between students and dentists (p=0.02) and students and professionals (p<0.001). There are no differences between dentists and specialists (p=0.07). ^4^Statistically significant differences regarding the correct diagnosis between dentists and specialists (p=0.001). There are no differences between the other groups.
